# Amine-modified nanoplastics promote the procoagulant activation of isolated human red blood cells and thrombus formation in rats

**DOI:** 10.1186/s12989-022-00500-y

**Published:** 2022-09-14

**Authors:** Eun-Hye Kim, Sungbin Choi, Donghyun Kim, Han Jin Park, Yiying Bian, Sang Ho Choi, Han Young Chung, Ok-Nam Bae

**Affiliations:** 1 College of Pharmacy Institute of Pharmaceutical Science and Technology, Hanyang University, Ansan, 15588 Republic of Korea; 2 School of Public Health, China Medical University, Shenyang, 110122 China; 3 National Research Laboratory of Molecular Microbiology and Toxicology, Department of Agricultural Biotechnology, and Center for Food and Bioconvergence, Seoul National University, Seoul, 08826 Republic of Korea

**Keywords:** Microplastics, Nanoplastics, Cardiovascular systems, Red blood cells, Procoagulation activity

## Abstract

**Background:**

Microplastics (MPs) and nanoplastics (NPs) formed from decomposed plastic are increasing environmental threats. Although MPs and NPs exposed through various routes enter the systemic circulation, the potential toxicity of those is largely unknown. We investigated whether polystyrene NPs (PS-NPs) promote the coagulation activity of red blood cells (RBCs).

**Results:**

We tested several types of PS-NPs using human RBCs and found that amine-modified 100 nm PS-NPs were the most potent. We measured the uptake of PS-NPs using flow cytometry and confocal microscopy. Electron microscopy revealed morphological changes of RBCs by PS-NPs. PS-NPs induced the externalization of phosphatidylserine, generation of microvesicles in RBCs, and perturbations in the intracellular microenvironment. PS-NPs increased the activity of scramblases responsible for phospholipid translocation in RBCs. PS-NPs modulated the functional interaction to adjacent tissues and coagulation cascade, enhancing RBC adhesion and thrombin generation. Our observations in human RBCs were consistent with those in isolated rat RBCs, showing no inter-species differences. In rat venous thrombosis models, the intravenous administration of PS-NPs enhanced thrombus formation.

**Conclusion:**

Amine-modified PS-NPs induce the prothrombotic activation of RBCs causing thrombus formation. We believe that our study will contribute to understanding the potential toxicity of amine-modified polystyrene particles in blood cells and cardiovascular systems.

**Supplementary Information:**

The online version contains supplementary material available at 10.1186/s12989-022-00500-y.

## Background

The number of plastic products manufactured and disposed of every year worldwide is increasing [1–3]. Discarded plastics may accumulate in various places and decompose into smaller sizes following exposure to light, ultraviolet radiation, embrittlement, biological factors, and sea-salt aerosol formation [4–6]. Decomposed plastics may be divided into various sizes, such as microplastics (MPs) (0.1 μm–5 mm) and nanoplastics (NPs) (≤ 0.1 μm) and accumulate a variety of functional groups (non-functionalized, carboxyl, and amine) on their surfaces [7–9]. Polystyrene is a common plastic used in plastic products because of its high durability and chemical inertness over a wide range of temperatures. Polystyrene is used in a variety of thermal insulation, packaging, and disposable products, suggesting that polystyrene may enter the environment through a variety of routes [10–12].

Because of the increase in the number of plastics that accumulate in the environment and are not easily removed, research on the effects of various types of plastics on the environment and human cells has been conducted [13, 14]. Decomposed plastics exist in environments such as soils, fresh water, rivers, and oceans and contribute to environmental pollution [15–17]. Decomposed plastics in the environment may confer harmful effects on organisms [18–20]. They may cause dysfunction and toxicity in a variety of human-derived cells such as intestinal, liver, lung, and immune cells [21–25]. Although studies are ongoing, little is known about the risks of plastics in humans. Additionally, since decomposed plastics of MPs and NPs may be contaminated with xenobiotics such as heavy metals and persistent organic pollutants through surface interactions, further research on their effects on health is important [5, 26, 27]. In addition to xenobiotics, various microorganisms can be attached to MPs and NPs. Plastic with microorganisms bound to these surfaces can transport microorganisms, including pathogens, into the environment as well as the human body [28, 29].

MPs and NPs enter the body through diverse pathways. Depending on their size, MPs and NPs translocate from primary target organs, such as the intestine, to other tissues, and particles < 10 μm migrate into the bloodstream [12, 30–32]. Particulate matter (PM) can contribute to an increased risk of cardiovascular disease [33–35]. PM_2.5_, PM with a diameter ≤ 2.5 μm, plays an important role in biological mechanisms such as inflammation and hypercoagulability, thereby influencing the cardiovascular system [36–38].

Xenobiotics, including chemical and particulate contaminants entering the bloodstream, come into contact with various types of blood cells. Red blood cells (RBCs), the main component of blood, play an important role in cardiovascular homeostasis by regulating vascular function and stiffness. Although RBCs are inert, they play a role in promoting the coagulation cascade and thrombus formation in venous thrombosis [39, 40]. The externalization of phosphatidylserine (PS) to the outer leaflets of the lipid bilayer and intracellular calcium in RBCs are key in coagulation cascade and adhesion to blood vessels [41–44]. Variations in intracellular calcium content cause the activation of scramblase, which may result in the exposure of PS to the outer membrane leaflet [45]. Micro- and nanoparticle aggregates (~ 0.2 μm) can attach to the surface of RBCs [46, 47]. The size and surface of MPs and NPs play an important role in the aggregation and adhesion of RBCs [48–50].

In this study, we investigated the effects of polystyrene NPs (PS-NPs) on freshly isolated human RBCs. We examined the uptake of PS-NPs into RBCs and analyzed the morphological alterations in RBCs. Exposure to PS-NPs induced membrane changes via PS externalization and microvesicle (MV) generation in RBCs. The underlying mechanisms of intracellular calcium levels and scramblase activity were measured. These changes induced functional prothrombotic events, including thrombin generation and endothelial adhesion. The in vivo relevance of prothrombotic activation was determined using a rat in vivo venous thrombosis model after the intravenous injection of PS-NPs.

## Results

### Hemolytic effects of polystyrene plastic particles on human RBCs

**Fig. 1:**
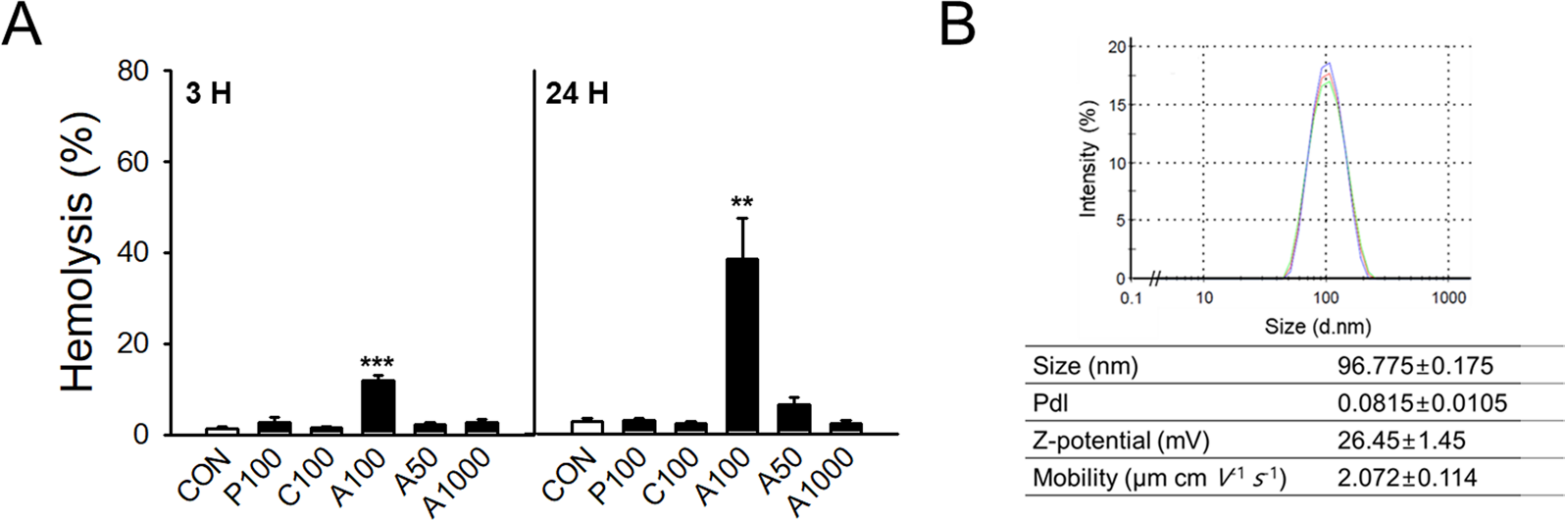
Screening for the effect of polystyrene on size and surface in human RBCs. We treated human RBCs with 100 µg/mL of amine-modified polystyrene 50 nm (A50), 100 nm (A100), and 1000 nm (A1000) in size; 100 nm plain polystyrene (P100); and 100 nm carboxyl-modified polystyrene (C100). Subsequently, hemolysis was evaluated (*n* = 4–6) (**A**). The size distribution of PS-NPs in Ringer’s solution detected using DLS (**B**). Data are presented as the mean ± SE. **p < 0.01, ***p < 0.001 versus control (CON)

The size and functional groups attached to the surface of plastic particles are major factors affecting their behavior or toxicity [51–53]. To investigate the effects of polystyrene MPs and NPs, we tested several types of PS-NPs and selected the most potent form of plastic particles using freshly isolated human RBCs. RBCs were exposed to 100 nm-sized plain, carboxyl-modified, or amine-modified polystyrene particles (P100, C100, or A100, respectively), or amine-modified polystyrene particles 50, 100, or 1000 nm in size (A50, A100, or A1000, respectively). The extent of hemolysis was determined spectrophotometrically based on the released level of hemoglobin at 3 h or 24 h after particle exposure to RBCs at a concentration of 100 µg/mL (Fig. 1A). Among the polystyrene particles tested, A100 showed a statistically significant and the greatest hemolysis at both 3 h and 24 h. We selected A100 to conduct the subsequent experiments and characterized A100, which has been referred to as the PS-NP throughout the results and discussion of this report to represent polystyrene NPs. To characterize the A100 PS-NPs, the size and zeta potential were measured using dynamic light scattering. The average peak size by intensity frequency of the PS-NP was 96.775 ± 0.175 nm, and the calculated zeta potential was 26.45 ± 1.45 mV as suspended in Ringer’s solution (Fig. 1B).

### Observation of cellular uptake of PS-NPs by human RBCs

**Fig. 2:**
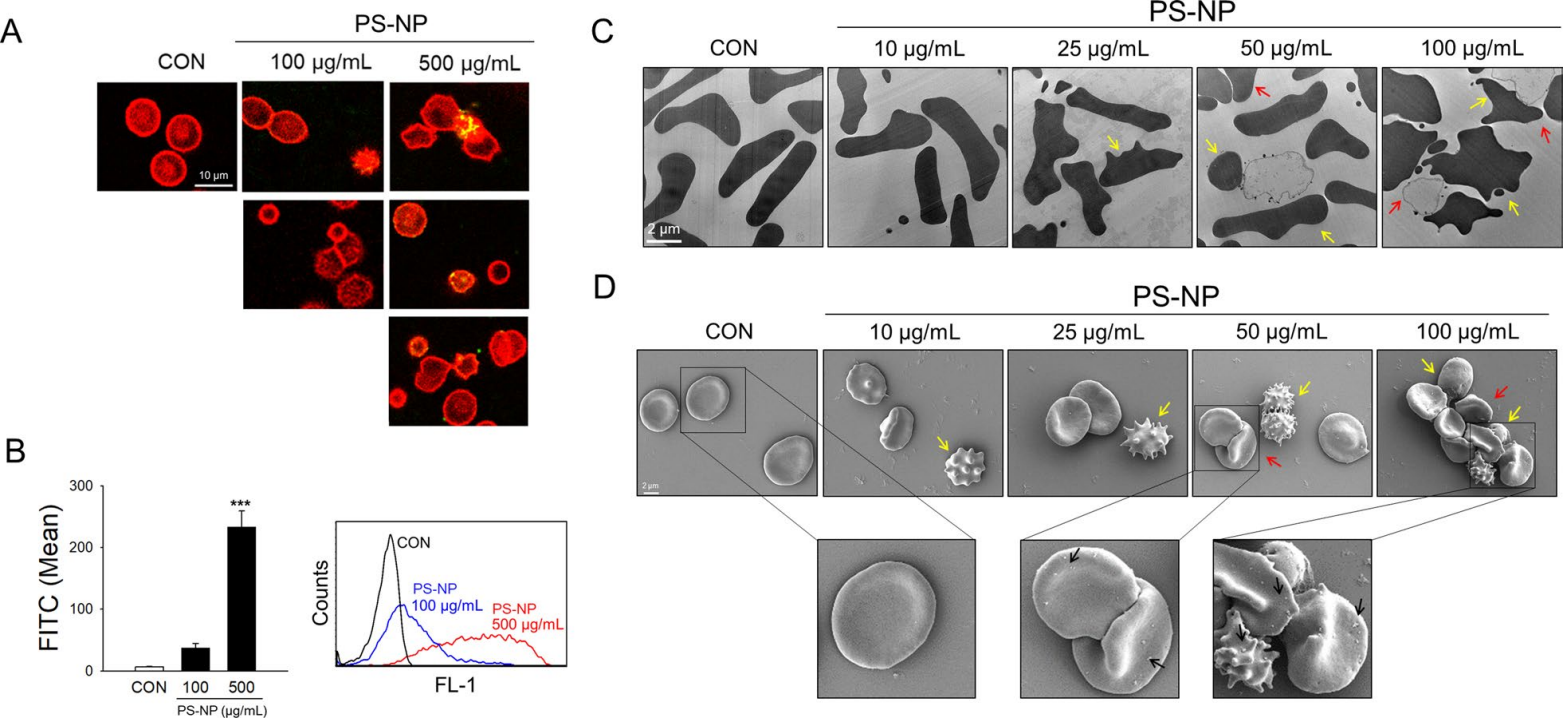
Uptake and morphological changes in human RBCs by PS-NPs. We confirmed the uptake of PS-NPs in RBCs using confocal microscopy (*n* = 4) (**A**) and flow cytometry (*n* = 4) (**B**). Changes in the shape of RBCs by PS-NPs were observed using TEM (*n* = 4) (**C**) and SEM (*n* = 4) (**D**). The yellow arrows indicate spiny RBCs, called echinocytes, and spherocytes. Aggregation occurs by PS-NPs indicated by the red arrows. The black arrows show PS-NPs attached to the surface of RBCs. Data are presented as the mean ± SE. ***p < 0.001 versus control (CON)

Confocal microscopy was used to observe the uptake and localization of PS-NPs in RBCs. Green fluorescence dot signals from PS-NPs were observed in RBCs identified with red fluorescence in PS-NP-exposed RBCs for 3 h (Fig. 2A). Along with the PS-NP uptake, changes in the morphology or aggregation of RBCs were observed, and the green dots were localized within the RBCs as well as the RBC aggregates. Flow cytometric analysis showed an increase in PS-NP fluorescence in RBCs exposed to PS-NPs at 100 or 500 μg/mL in a dose-dependent manner, indicating that PS-NPs were significantly taken up into or attached to RBCs (Fig. 2B).

### Morphological changes in human RBCs by PS-NPs

We observed the morphological changes in RBCs using confocal microscopy with fluorescence (Fig. 2A). The detailed shape changes in RBCs from discocytes to echinocytes and spherocytes were further observed using TEM and SEM, following exposure to PS-NPs. Although we used a higher concentration of PS-NPs (100 or 500 μg/mL) to clearly visualize and quantify the uptake of PS-NPs (Fig. 2A, B), we applied a lower concentration range of PS-NPs (up to 100 μg/mL) to focus on the sensitive morphological changes in RBCs. The shape of discocytic RBCs changed to echinocytic and spherocytic RBCs following PS-NP exposure (yellow arrows), and RBC aggregates (red arrows) were observed at higher concentrations of PS-NPs (greater than 50 μg/mL) 3 h after exposure, as observed using TEM (Fig. 2C) and SEM (Fig. 2D). Notably, in SEM analysis, PS-NPs attached to the surface of RBCs were observed in a concentration-dependent manner on the surface of echinocytes, spherocytes, and aggregated RBCs (Fig. 2D).

### PS-NP-induced hemolysis, PS externalization and MV generation in human RBCs

**Fig. 3:**
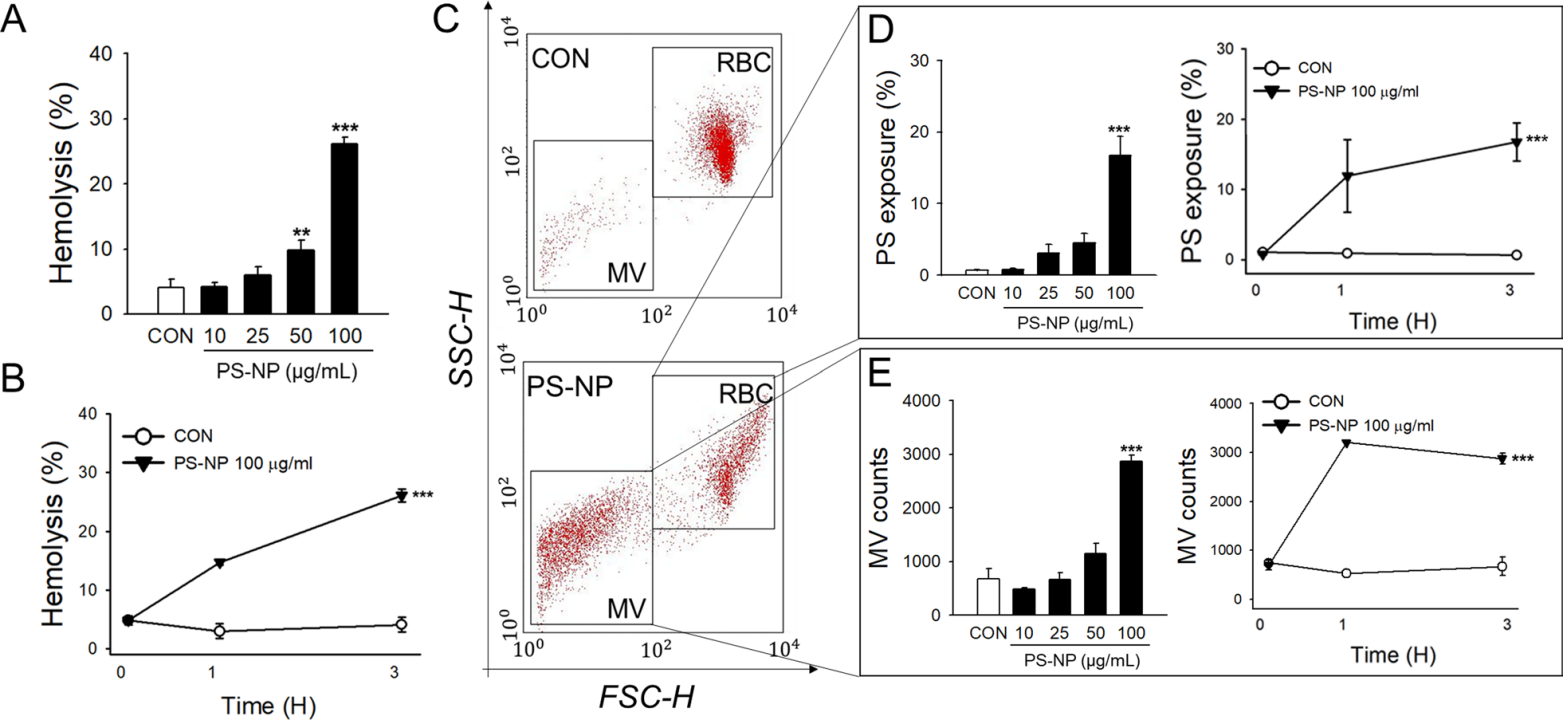
Effects of PS-NPs on human RBCs. Various concentrations of PS-NPs were treated with human RBCs for 3 h (**A**). Hemolysis was evaluated spectrophotometrically at a wavelength of 540 nm, and a time-course study was conducted with 100 µg/mL of PS-NPs (*n* = 5) (**B**). PS exposure and MV generation were measured using flow cytometry (**C**). Concentration-dependent increase at 3 h and the time-course of PS exposure (**D**) and MV generation (**E**) were determined (*n* = 5). Data are presented as the mean ± SE. **p < 0.01, ***p < 0.001 versus control (CON)

Observation of the RBC morphology showed that PS-NPs significantly affected the RBC membrane integrity. To determine the effective concentration- and time windows of PS-NPs to induce RBC changes, we first measured PS-NP-induced hemolysis of human RBCs. After 3 h of treatment, we found concentration-dependent and significant hemolysis by PS-NPs at concentrations greater than 50 µg/mL (Fig. 3A). The extent of hemolysis was 25.97 ± 3.04% with 100 µg/mL of PS-NPs. The hemolytic effect of PS-NPs (100 µg/mL) was exposure time-dependent, as shown in Fig. 3B. Hemolysis is often associated with membrane changes in RBCs, such as the externalization of PS and generation of MVs, which contribute to the procoagulant activation of RBCs [54]. Further, we investigated whether PS-NPs induced PS externalization and MV generation in human RBCs using flow cytometry. As observed by the shift in RBC distribution in forward- and side-scattering analysis (Fig. 3C), the subpopulation of RBC MVs, the sizes of which were smaller than 1 μm, was significantly increased by 100 µg/mL of PS-NPs. PS-NP treatment significantly induced PS externalization to the outer membrane of RBCs in a concentration- and time-dependent manner (Fig. 3D). MV generation was also significantly increased following PS-NP treatment (Fig. 3E). The loss of phospholipid asymmetry and membrane blebbing are well-associated phenomena with morphological changes in RBCs [55], and we observed these consistent effects on membrane alteration and morphological changes in human RBCs exposed to PS-NPs.

### Intracellular mechanisms responsible for the membrane changes in RBCs by PS-NPs

**Fig. 4:**
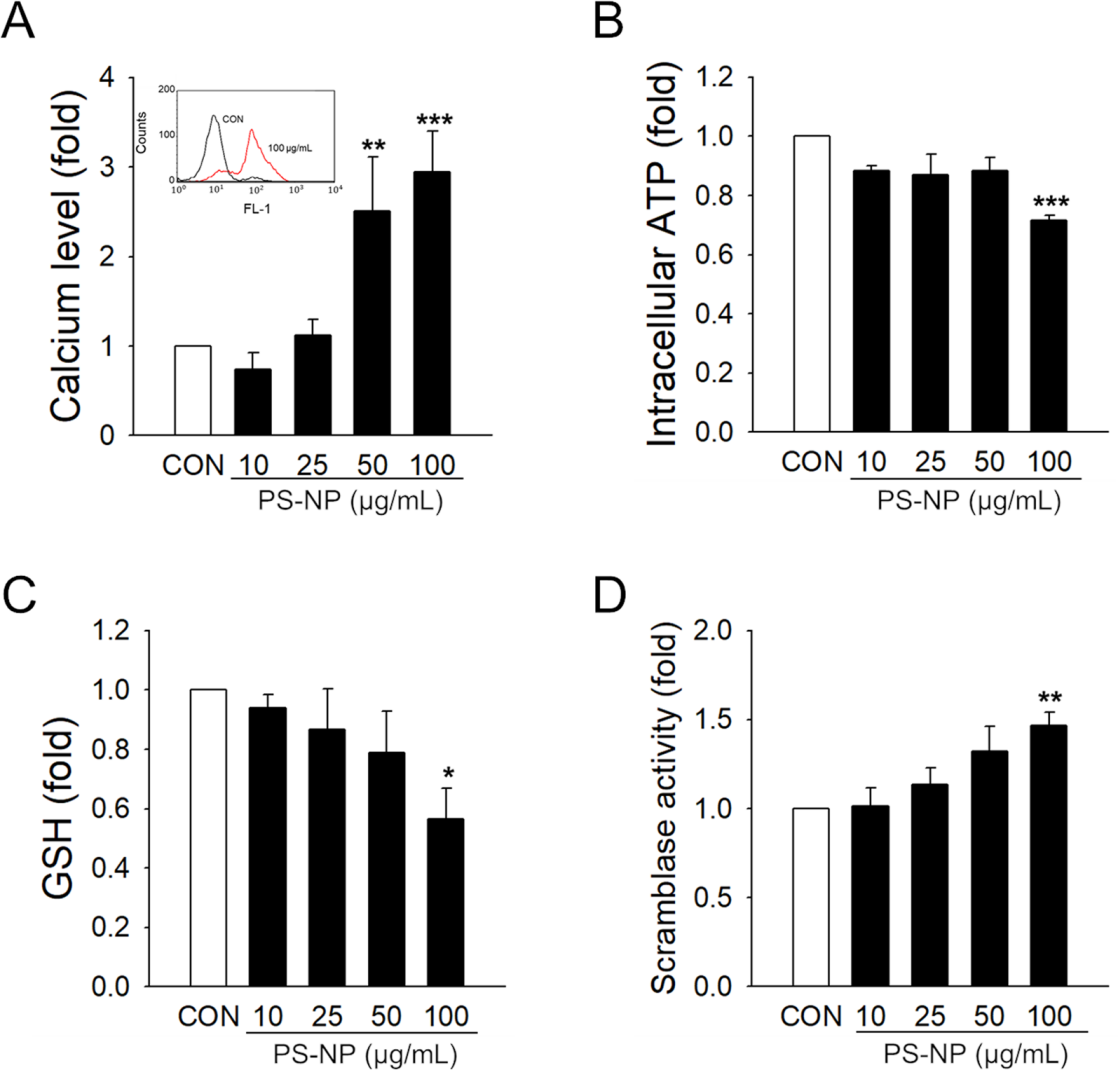
Measurement of the mechanism underlying coagulation activation in human RBCs. After RBCs were treated with various concentrations of PS-NPs for 3 h, intracellular calcium levels (*n* = 5) (**A**) were detected. Depletion of intracellular ATP levels (*n* = 4) (**B**) and GSH levels (*n* = 6) (**C**) were observed after RBCs were exposed to PS-NPs. Scramblase activity was determined by measuring the translocation 10 min after C6-NBD-PC treatment (*n* = 7) (**D**). Data are presented as the mean ± SE. *p < 0.05, **p < 0.01, ***p < 0.001 versus control (CON)

The maintenance of membrane integrity in RBCs is tightly regulated by enzymes responsible for membrane asymmetry, such as scramblase [56], and several intracellular factors, including the levels of intracellular calcium, ATP, and GSH [57–59]. To understand the mechanisms underlying PS externalization and MV generation in RBCs by PS-NPs, we measured the changes in intracellular mediators. Intracellular calcium levels were significantly increased in a concentration-dependent manner in the PS-NP-treated RBCs (Fig. 4A). PS-NP treatment significantly induced the depletion of intracellular ATP (Fig. 4B) and GSH, the representative intracellular antioxidant, in RBCs (Fig. 4C). Two distinct enzyme systems are responsible for membrane phospholipid asymmetry. Scramblase, an enzyme activated by increased intracellular calcium, contributes to the bidirectional scrambling of phospholipids in the inner and outer membranes [60, 61]. We observed that the activity of scramblase was significantly increased by PS-NPs in RBCs (Fig. 4D), suggesting that the activation of scramblases by PS-NPs may contribute to disturbances in membrane asymmetry.

### Prothrombotic effects of PS-NPs on human RBCs

**Fig. 5:**
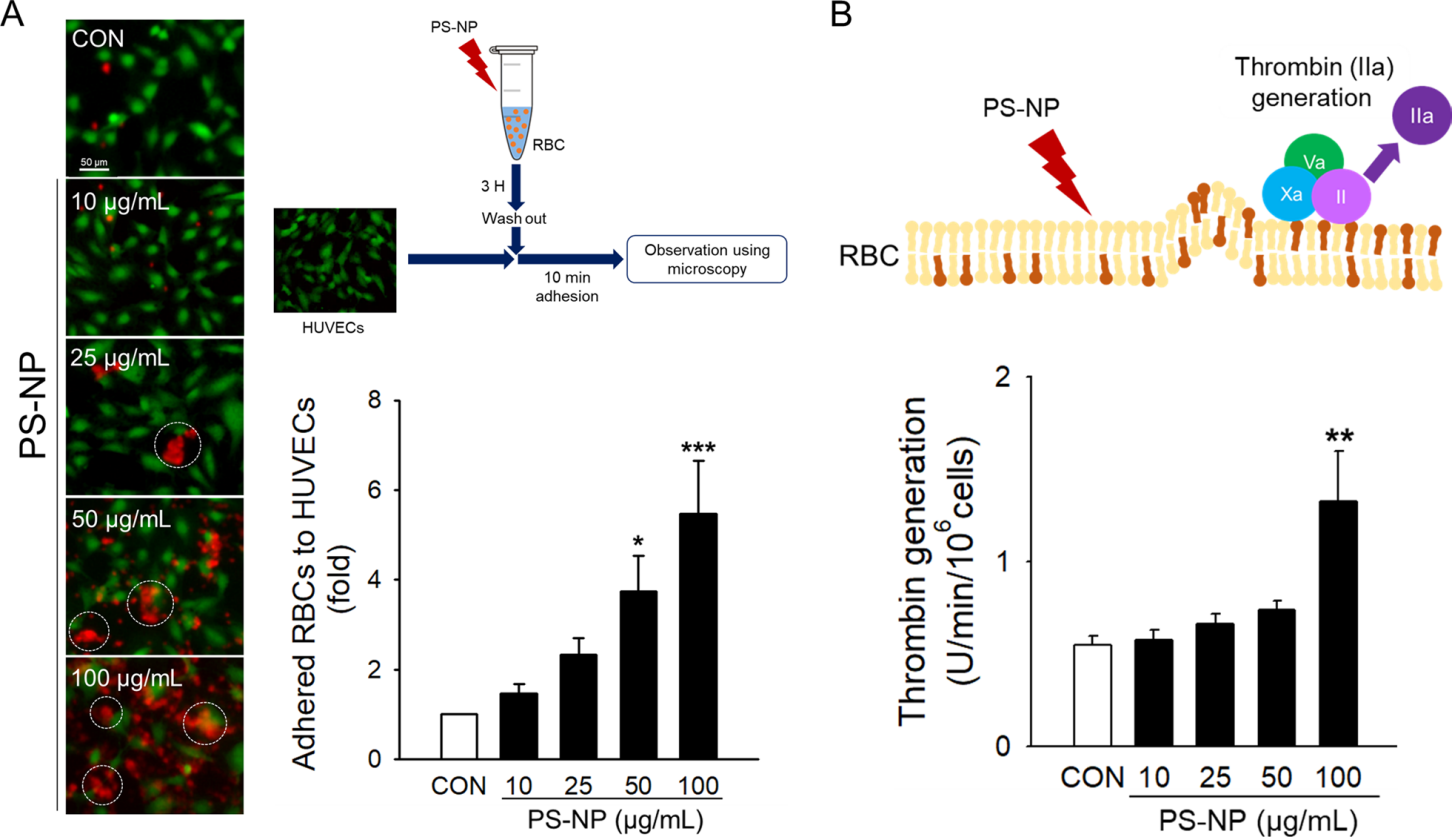
Effects of PS-NPs on the adhesion of human RBCs to human umbilical vein endothelial cells (HUVECs) and thrombin generation. **A** Adhesion of RBCs incubated with PS-NPs to HUVECs was observed using fluorescence microscopy (*n* = 4). The signal of endothelial cells is green fluorescence and that of RBCs is red fluorescence. The white circles indicate the aggregated RBCs. **B** Thrombin generation was determined at the various concentrations of PS-NPs at a wavelength of 405 nm (*n* = 6). Data are presented as the mean ± SE. *p < 0.05, **p < 0.01, ***p < 0.001 versus control (CON)

While remnant RBCs are not actively involved in hemostasis and thrombosis, dysregulated RBCs with externalized PS and generated MVs may efficiently participate in the process of coagulation [62], as well as adhesion to blood vessels [63, 64]. To examine the pathological role of PS-NP-induced RBC changes, we measured the prothrombotic activation of RBCs using the two representative functional parameters of adhesion to ECs and procoagulation. The adhesion of RBCs to HUVECs was analyzed using fluorescence microscopy. Treatment with PS-NPs significantly increased the adhesion of RBCs (red fluorescence) to HUVECs (green fluorescence) in a concentration-dependent manner (Fig. 5A). Notably, aggregated RBCs adhered to endothelial cells were observed (white circle). The procoagulant activity of RBCs was examined by measuring enhanced thrombin generation, a key step in the blood coagulation cascade [65]. RBCs exposed to PS-NPs showed significantly increased thrombin generation (Fig. 5B).

### In vivo assessment of PS-NP-induced prothrombotic effects

**Fig. 6:**
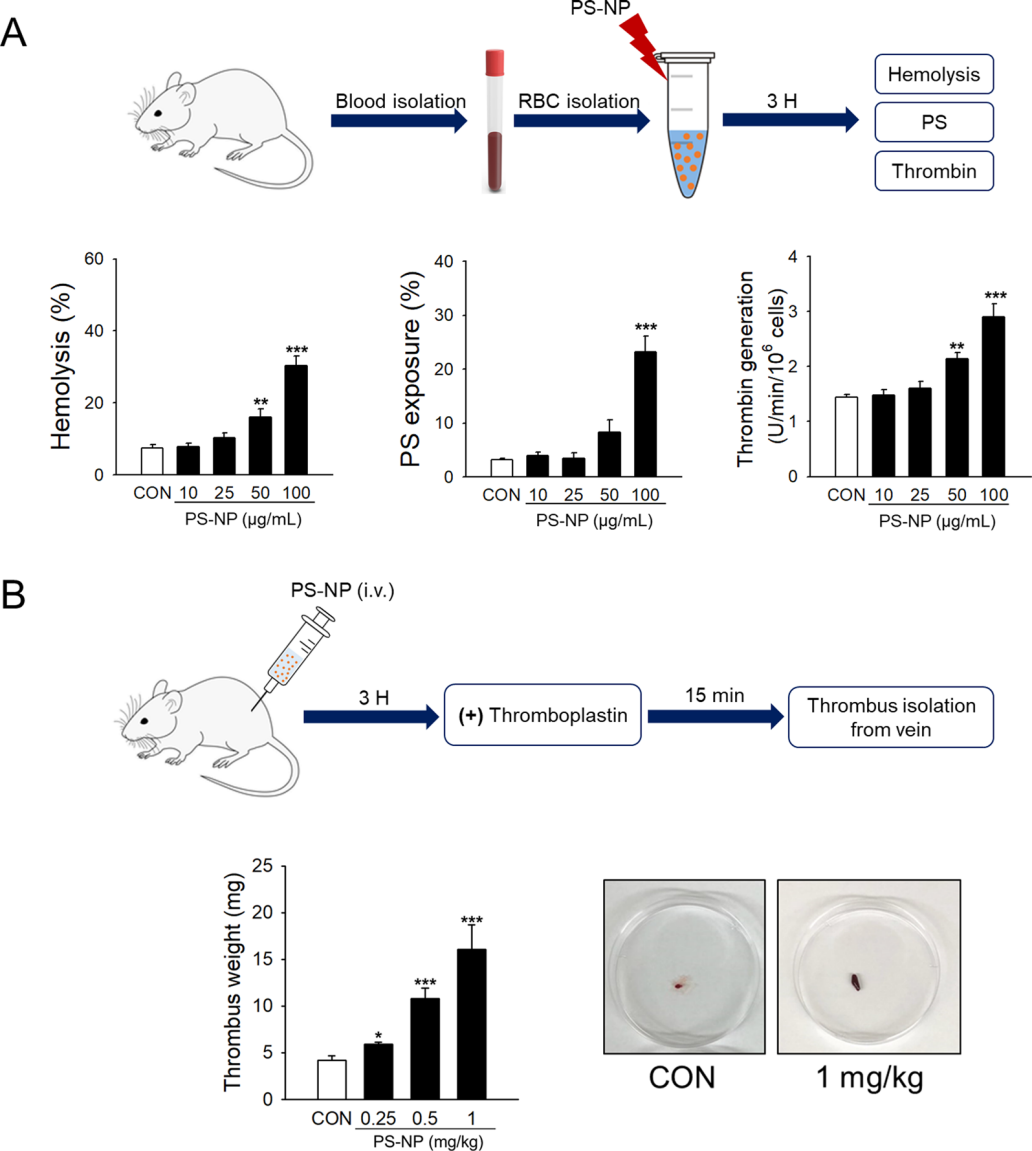
Effects of PS-NPs on rat RBCs in vitro and rat venous thrombosis model in vivo. **A** Freshly isolated rat RBCs were treated with various concentrations of PS-NPs for 3 h. Hemolysis (*n* = 6), PS exposure (*n* = 4), and thrombin generation (*n* = 6) were measured as described in the methods. **B** Three hours after intravenous injection of saline (control) or PS-NPs (0.25, 0.5, or 1 mg/kg), thrombus formation was induced by the infusion of thromboplastin in a rat venous thrombosis model. We showed the different sizes of thrombus from a rat vein (*n* = 4–8). Data are presented as the mean ± SE. *p < 0.05, **p < 0.01, ***p < 0.001 versus control (CON)

Further, we evaluated the in vivo relevance of our observations using rat venous thrombosis models. Prior to the rat in vivo study, we examined whether there were any species differences between humans and rats in PS-NP-induced RBC toxicity. We investigated the hemolytic effects of PS-NPs on freshly isolated rat RBCs. Consistent with human RBCs, a significant concentration-dependent increase in hemolytic activity was observed in rat RBCs after exposure to PS-NPs (Fig. 6A, left panel). Significant increases in PS externalization to the outer membrane and thrombin generation were also observed in rat RBCs after PS-NP treatment for 3 h (Fig. 6A, middle and right panels, respectively). To induce venous thrombosis in rats, thromboplastin was injected into the rat abdominal vein, and intravenous administration of PS-NPs 3 h before thromboplastin stimulation increased thrombus formation (Fig. 6B). A significant dose-dependent enhancement of thrombus weight by PS-NPs was observed even at the lowest dose tested (0.25 mg/kg).

## Discussion

**Fig. 7:**
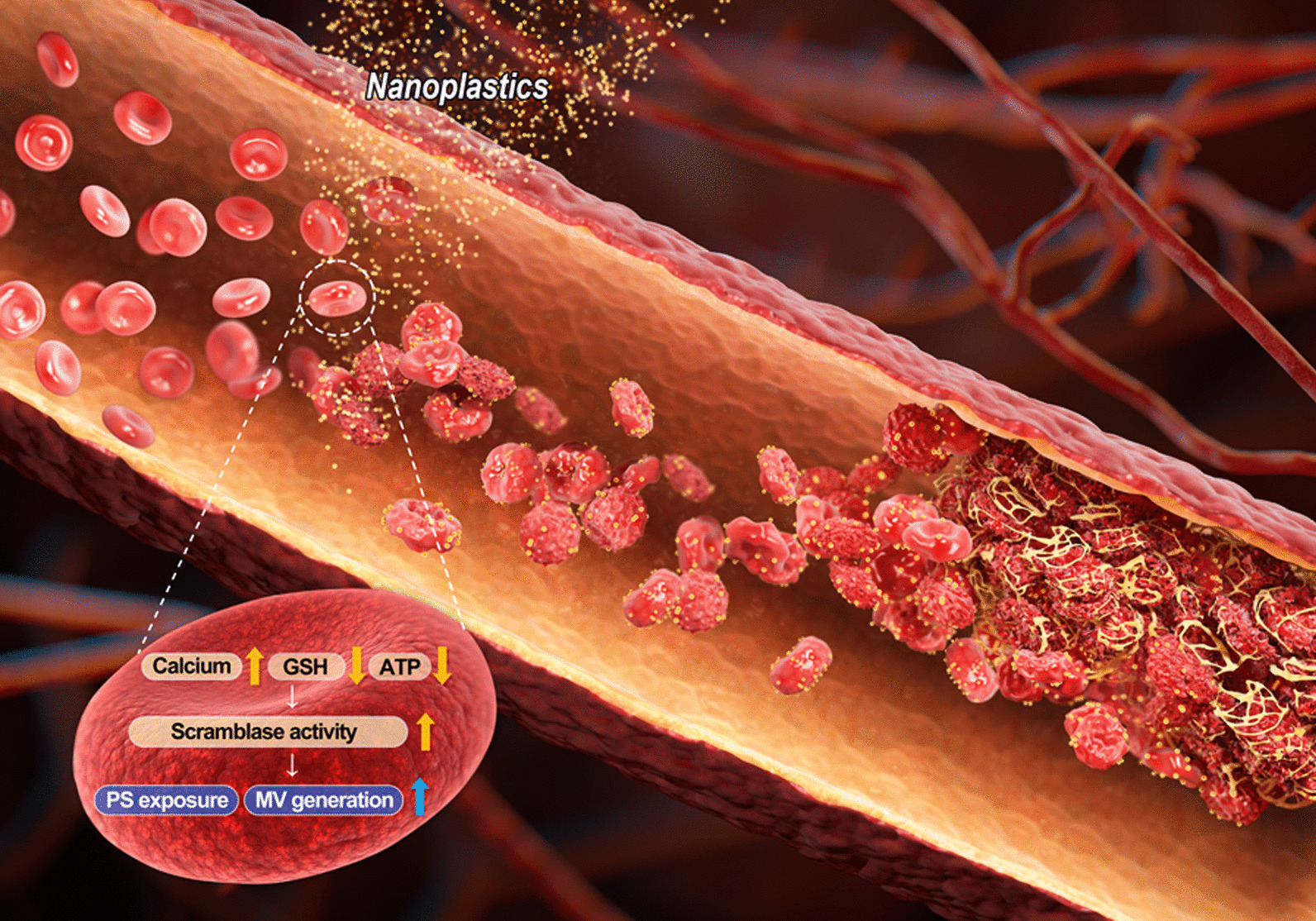
Scheme for the effect of PS-NPs on the procoagulant activity of RBCs contributing to thrombosis. PS-NPs entering the body are exposed to RBC during circulation. PS-NPs are attached to the RBC surface and induce morphological change. Changes in the intracellular environment, such as increased levels of intracellular calcium and decreased levels of glutathione and ATP, are observed in exposed RBCs, resulting in increased scramblase activity. These alterations on RBCs lead to increased phosphatidylserine (PS) exposure and microvesicle (MV) formation by PS-NPs. PS-NPs affect the adhesion of RBC to endothelial cells (EC) and promote procoagulant activity, increasing thrombus formation in blood vessels

In the current study, we demonstrated that PS-NPs promote procoagulant activation in human and rat RBCs. PS-NPs were selected as the most potent form among the various plastic particles in human RBCs. We demonstrated the uptake of PS-NPs into human RBCs and the morphological and functional changes in RBCs even at non-hemolytic concentrations. PS-NPs significantly increased hemolysis, PS externalization, and MV generation, resulting in enhanced thrombin generation and adhesion to HUVECs. The levels of several intracellular mediators, including intracellular calcium, GSH, and ATP levels, were affected, leading to the activation of scramblase. Notably, thrombus formation was significantly increased in rats administered PS-NPs intravenously (Fig. 7).

In addition to being an emerging threat to the environment, MPs and NPs have also been reported to be ingested by living organisms, including marine species, plants, and animals [66, 67]. Along with the accumulating research outcomes of ecotoxicology, potential adverse health effects on humans have been extensively studied [4, 22, 68]. In several experimental in vitro and in vivo models, MPs and NPs induce various effects, such as inflammatory and immune responses [69, 70]. MPs and NPs entering the body circulate across cell membranes, leaving most tissues and organs exposed [31, 71, 72]. Such exposure may cause oxidative stress and inflammation and is linked to the risk of detrimental effects, such as cardiovascular and respiratory diseases [73]. While concerns regarding MPs and NPs burden on the environment and human health have been rapidly increasing, studies identifying the adverse health effects of these plastics remain limited. In this study, we investigated the effects of MPs and NPs on human blood cells, which are continuously exposed to circulating pollutants on in vivo absorption.

Many studies have focused on the toxicity of MPs and NPs in the respiratory or gastrointestinal (GI) tract, where inhalation and ingestion exposure occurs [74]. These particles cross the epithelial barriers of the respiratory and GI tracts [30], and the smallest plastic particles (≤ 100 nm) pass through the cell membrane barriers and access all organs, including the placenta and brain [31, 32, 72]. Indeed, MPs and NPs entering the body can migrate to the bloodstream, where they translocate and distribute to various organs. Blood cells and vascular systems are the migratory path for the plastic particles; however, they may also be the target tissues during circulation. A previous study investigated the effects of polystyrene particles on peripheral blood mononuclear cells and RBCs and found significant hemolysis of RBCs using 460 nm and 1 µm products [12]. In the present study, we focused on functional alterations in RBCs (i.e., procoagulant activation), at a smaller size and lower concentration of PS-NPs. In the rat venous thrombosis model, intravenously administered PS-NPs significantly enhanced thrombus formation (Fig. 6B), providing important evidence that plastic particles circulating in the bloodstream may contribute to cardiovascular and thrombotic diseases. Notably, our in vivo study matched well with previous reports where ultrafine particles (UFPs) are associated with thrombosis in rats and hamsters [75, 76]. The authors used 60 nm amine-modified polystyrene particles as representative UFPs, with a similar in vivo dosage compared to our study. The enhanced thrombus formation was consistently observed in previous studies and our current study, and we show that the alterations of RBCs would play critical roles in this phenomenon.

There is accumulating evidence showing that the exposure levels of MPs and NPs to living organisms are rapidly increasing [77]. Due to the high environmental burden, people can be exposed to MPs and NPs throughout a lifetime, posing a concern for chronic and cumulative exposure. It is reported that the general human population ingests about 5 g of plastics including polystyrenes per week, about 21 g per month, and more than 250 g per year, mainly through food consumption and drinking water [78, 79], and approximately 0.01 g/kg BW/day of plastics are exposed to humans. In addition to the ingestion, polystyrene can enter the body through inhalation and dermal contact, resulting in aggregate exposure [32]. In this study, we used 0.25–1 mg/kg of single intravenous administration of polystyrene particles to rats and 10–100 µg/mL to isolated RBCs. The in vivo and in vitro concentration ranges were correlated under the assumption that the intravenously administered PS-NP was mainly distributed to the blood compartment, posing blood cells as the main target organ. It should be noted that the exposure duration in our experimental system (several hours) is relatively short compared to the real exposure scenario in the environment (throughout the lifetime). Nevertheless, the absorbed and the blood circulating levels of MPs and NPs have not been precisely reported yet, making it difficult to estimate the relevant human internal dosages of MPs and NPs. A recent study provided a probabilistic exposure simulation for children and adults using the physiologically-based pharmacokinetic models, suggesting that 184 ng/capita/day as median intake levels for MPs (1–5000 µm), and 40.7 ng/capita as accumulated tissue levels for 1–10 µm particles until age 70 for adults [78]. Further studies on the absorption and behavior of MPs would be required to study the health effects of these plastic particles.

Damage to blood cells may contribute to cardiovascular toxicity [50, 80]. RBCs are considered to play passive role, such as in the delivery of oxygen, but have recently been increasingly recognized to play important roles in pathological conditions. Abnormal RBCs are associated with thrombotic diseases, such as venous thrombosis. In venous thrombosis, abnormal RBCs adhere to the vessel wall and contribute to procoagulant activity, which is an important pathological feature [81, 82]. As shown in Fig. 5, adhesion to vein endothelial cells and thrombin generation was significantly increased by PS-NPs, suggesting that plastic particles may promote thrombotic disease through the adhesion and procoagulant activity of RBCs. In addition, hemolysis and thrombus formation attributed to exposed PS and generated MVs are important in thrombotic disease. MVs, also called RBC-derived microparticles, contain procoagulant proteins that enhance coagulation through both the tissue factor and coagulation cascades [83]. Therefore, RBC MVs contribute to venous thrombosis and hemolytic activity [84]. Notably, recent studies have revealed the procoagulant activation of RBCs by exogenously introduced circulating contaminant particles, including silver, silica, titanium, and zinc oxide (from 50 to 2500 µg/mL), suggesting that these circulating particles may disturb RBC membrane integrity and contribute to thrombus formation and hemolytic conditions [85–89]. Physical toxicity as particles may also have contributed to the PS-NP-induced RBC toxicity observed in our study; however, we found functional and morphological alterations in RBCs at lower concentrations of PS-NPs (from 10 to 100 µg/mL), specifically by amine-modified forms. Notably, exposure to particle contaminants such as airborne particulate matter (PM 2.5 and PM 10) or bioaerosols, which circulate in the blood and come into contact with RBCs during absorption and distribution, may be associated with the prevalence of cardiovascular diseases, including thrombosis [90–92]. Due to the complexity of particle composition and characteristics, it may be difficult to generalize the role of particles in the thrombotic risk. In line with the present observations, the contribution of RBCs to these particle-associated thrombotic conditions requires further attention.

Maintenance of the intracellular levels of essential mediators such as calcium, ATP, and thiol is critical for membrane asymmetry and integrity [93–95]. As shown in Fig. 4, PS-NPs significantly altered the balance of these intracellular mediators, consequently leading to alterations in the activities of enzymes responsible for membrane integrity. These findings are consistent with those of previous studies, where an increase in intracellular calcium concentration and reduction in ATP are involved in the procoagulant activation of RBCs [41, 65]. Interestingly, changes in intracellular calcium levels in RBCs play critical roles in the membrane composition and rheological properties, as well as the redox state and RBC clearance [57, 96]. The dysregulation of calcium levels in RBCs is associated with several pathological states of sickle cell disease, thalassemia, and thrombotic complications [40, 97, 98]. Another key mediator of PS-NP toxicity in RBCs is ATP. RBCs are highly deformable structures with delicately regulated membrane cytoskeleton interactions, which are ATP-dependent mechanisms [58]. The level of ATP is a key determining factor of stiffness in stored RBCs [99] or in aged and senescent RBCs [100]. The precise mechanisms by which PS-NPs affect the intracellular mediators of calcium and ATP must be elucidated, and the impact of abnormal calcium and ATP regulation by PS-NPs in RBCs may need to be further considered for their toxicological and pathological significance.

Plastic particles exhibit various toxicities depending on the functional groups attached to their surface [11, 101]. As shown in Fig. 1, the different functional groups attached to the surface of PS-NPs induced RBC hemolysis at different potencies, demonstrating that the amine group modification may be important to initiate RBC alterations. Among the particles with the same amine modification, those with a size of 100 nm caused the most potent hemolysis of human RBCs. Consistent with the hemolysis, amine-modified 100 nm polystyrene particles displayed the most significant and highest extents of PS externalization, MV formation, and thrombin generation among several forms tested with different functional groups and sizes (Additional file 1: Fig. S1). These findings are consistent with previously reported findings wherein amine-modified plastic particles showed more potent activities in apoptosis, lysosomal acidification, and loss of mitochondrial membrane potential in different organ cell types, such as lung, liver, and kidney cells and macrophages [101]. The precise mechanisms underlying the higher activity of amine-modified forms remain unclear; however, Wang et al. suggested that dysfunctional lysosomes and deregulated autophagy may contribute to the adverse effects of amine-modified plastic particles [102]. In addition to polystyrene, it has been reported that the amine groups on the surface are more effective in various particles such as dendrimers and zinc oxide nanorods [103, 104]. Future studies should investigate how the amine-modified PS-NPs enter RBCs and how amine modification contributes to membrane disturbances. Interestingly, amine-modified polystyrene particle-sized 50 nm (A50), which has a smaller size, a higher number of particles, and a bigger surface area compared to A100 at the same concentration, was not effective as A100, suggesting that the surface modification, as well as the size and the surface area, can be important determinants in the toxicity induced by particles.

Our study used spherical polystyrenes to confirm the toxicity of PS-NPs in RBCs. However, plastics exist in various forms in the environment, such as plastic fragments or fibers [105], and the shape we used does not completely represent the plastics that exist in the environment. The shape and groups attached to the surface affect the toxicity of plastics [106]. Fibers have more harmful effects on organisms such as zooplankton and Pacific mole crabs than those of other forms [107, 108]. The effects of plastics on humans have not yet been elucidated. Previously, most studies used spherical plastics, which do not fully represent the plastics forms that exists in the environment, so research on various types of plastics is also needed [109]. If research on the effects of plastics on humans is expanded in the future, it would be necessary to evaluate the effects of plastics of various shapes on RBCs and thrombotic diseases. Nevertheless, the present study evaluated the effect of NPs on blood cells, and it may be helpful to inform future studies of the human effects of MPs and NPs.

## Conclusion

Our study demonstrated that NPs, specifically amine-modified polystyrene particles 100 nm in size, significantly affect morphology and membrane asymmetry in human RBCs, promoting adhesion to endothelial cells and thrombin generation. In vivo rat models revealed that thrombus formation is significantly enhanced by the intravenous administration of plastic particles. We hope that our current study contributes to the understanding of the adverse health effects of plastic particles.

## Materials and methods

### Materials

Glutaldehyde solution, osmium tetroxide, and an adenosine triphosphate (ATP) bioluminescence assay kit were purchased from Sigma-Aldrich (St. Louis, MO, USA). Phycoerythrin-labeled monoclonal mouse anti-human CD235a and fluorescein isothiocyanate (FITC)-labeled annexin V (annexin V-FITC) were purchased from BD Biosciences (Bergen County, NJ, USA). Fluo-4 acetoxymethyl ester (fluo-4 AM) was purchased from Thermo Fisher Scientific (Rockford, IL, USA). The GSH-Glo™ glutathione (GSH) assay kit was purchased from Promega (Madison, WI, USA). 1-oleoyl-2-[6-[(7-nitro-2-1,3-benzoxadiazol-4-yl)amino]hexanoyl]-sn-glycero-3-phosphocholine (C6-NBD-PC) was purchased from Avanti Polar Lipids (Alabaster, AL, USA). Purified human thrombin was purchased from Merck Millipore (Burlington, MA, USA). Purified human prothrombin, factor Xa, and factor Va were purchased from Hematologic Technologies (Chittenden, VT, USA). S2238 was purchased from Chromogenix (Milan, Italy). Human umbilical vein endothelial cells (HUVECs) and the endothelial cell growth media (EGM) kit were purchased from Lonza (Basel, Switzerland). RecombiplasTin 2G was purchased from Instrumentation Laboratory Company (Middlesex, MA, USA). Amine-modified polystyrene sized at 50 nm (Cat #L0780), 100 nm (Cat #L9904), and 1000 nm (Cat #L1030) and polystyrene sized at 100 nm (Cat #LB1) were obtained from Sigma-Aldrich. Carboxylate-modified polystyrene sized at 100 nm (Cat #16688) was purchased from Polysciences (Warrington, PA, USA).

### Preparation of freshly isolated human RBCs

With approval from the Hanyang University Institutional Review Board (IRB 202012-001), human blood was obtained from healthy male donors (20–30 years of age) using an acid citrate dextrose vacutainer and 21-gauge needle (BD Biosciences) on the day of each experiment. After centrifugation at 200 × g for 15 min, the plasma and buffy coat were removed by aspiration. RBCs were washed twice with phosphate-buffered saline (PBS) and once with Ringer's solution. Isolated RBCs were resuspended in TBS buffer to a cell concentration of 5 × 10^7^ cells/mL, and the final CaCl_2_ concentration was adjusted to 1 mM before use.

### Measurement of the hemolytic activity of RBCs

After centrifugation of RBCs incubated with polystyrene, an aliquot of the supernatant was obtained. The extent of hemolysis was spectrophotometrically measured at a wavelength of 540 nm. RBCs lysed with 1% Triton X-100 were used as positive controls.

### Measurement of PS-NP uptake into RBCs

After incubation of PS-NPs (100 µg/mL and 500 µg/mL) with human RBCs for 3 h, the cells were centrifuged and washed once with Ringer’s solution. The uptake of PS-NPs was analyzed using flow cytometry and confocal microscopy. The fluorescence of PS-NPs taken up in RBCs was detected using a Guava EasyCyte 8 flow cytometer (Merck Millipore). For confocal microscopy, suspended RBCs were loaded onto a Lab-Tek™ 4-well Chambered Coverglass (Thermo Fisher Scientific) for 1 h. After washing with Ringer’s solution containing 2% bovine serum albumin (BSA), RBCs were incubated with PS-NPs. The RBCs were washed once again with Ringer’s solution after incubation and stained with anti-glycophorin-A-PE for 30 min. Fluorescence images were acquired and analyzed using a K1-Fluo confocal laser scanning microscope (Nanoscope Systems, Daejeon, Korea). The excitation and emission filters were set at 488 nm and 550–600 nm, respectively.

### Observation of morphological changes in RBCs

Morphological changes in RBCs were monitored using electron microscopy. For transmission electron microscopy (TEM) and scanning electron microscopy (SEM) observations, RBCs that were incubated with PS-NPs for 3 h were fixed with 2% glutaraldehyde solution for at least 1 h at 4 °C, and centrifuged at 5800 rpm, and washed with PBS. Post-fixation was performed with 1% osmium tetroxide for 30 min under a fume hood. After washing the RBCs twice with PBS, the samples were continuously dehydrated with 50%, 70%, 80%, 90%, and 100% ethanol; immersed in 100% ethanol; and stored at 4 °C. To observe RBCs using TEM, we used propylene oxide for transition every 10 min twice with dehydration of the samples under a fume hood. After infiltration with propylene oxide and Spurr’s resin (1:1) for 2 h and then with only Spurr’s resin in a desiccator overnight, the samples were treated with fresh Spurr’s resin for 2 h in a desiccator. The samples were maintained in an oven at 70 °C overnight for polymerization and observed using TEM (JEM 1010, JEOL). To confirm the SEM results, we used RBCs that were immersed in 100% ethanol for storage. The stored samples were dried and gold-coated, and the images were then observed using SEM (MERLIN Compact, Zeiss).

### Flow cytometric analysis of PS exposure and MV generation

Anti-glycophorin A-PE was used to identify RBCs, and annexin V-FITC was used to identify externalized PS in the outer membrane of the RBCs. Non-specific annexin V binding was determined in the presence of 2.5 mM of ethylenediaminetetraacetic acid (EDTA) instead of 2.5 mM of CaCl_2_. After treatment of polystyrene with RBCs, aliquots of the cell suspension were incubated with anti-glycophorin A-PE and annexin V-FITC for 30 min under dark conditions. Labeled samples were analyzed using the FACS Calibur flow cytometer (BD Biosciences) equipped with an argon-ion laser emitting at a wavelength of 488 nm. Data from 5000 events were collected and analyzed using Cell Quest Pro software.

### Measurement of intracellular calcium levels

To measure intracellular calcium levels, RBCs were incubated with 3 μM of Fluo-4 AM for 1 h at 37 °C under dark conditions. After centrifugation at 3000 rpm, the suspension was removed. The RBCs were incubated with PS-NPs for 3 h using Ringer’s solution containing CaCl_2_ to remove excess fluo-4 AM. The samples were diluted, and the fluorescence of intracellular calcium was measured using the Guava EasyCyte 8 flow cytometer.

### Measurement of intracellular ATP and GSH levels

After the RBCs were exposed to PS-NPs for 3 h, the intracellular levels of ATP and GSH were measured. ATP levels in RBCs were analyzed using diluted RBCs reacted with an ATP assay mix, following the protocol for the ATP bioluminescence assay kit (Sigma-Aldrich). To detect GSH levels, we followed the method described by the manufacturer of the GSH-Glo™ GSH Assay (Promega). After centrifugation, we removed the supernatant and added GSH-Glo reaction buffer. We centrifuged the suspended RBCs and transferred the supernatant to a new tube. Diluted samples were reacted with GSH-Glo reagent buffer and luciferin detection reagent under dark conditions. We measured the luminescence of ATP and GSH using the EnSpire (PerkinElmer).

### Detection of scramblase activity (phospholipid translocation)

The activity of scramblases was measured by incorporating fluorescence-labeled exogenous phosphatidylcholine (C6-NBD-PC). After 3 h of exposure with PS-NPs, RBCs treated with PS-NPs were incubated with 0.5 μM of C6-NBD-PC for 10 min at 37 °C. Aliquots from the cell suspension were removed at the indicated time intervals and stored on ice for 20 min in the presence or absence of 1% BSA. Pellets obtained after 1 min of centrifugation at 10,800 rpm were lysed using 1% Triton X-100, and fluorescence intensities were detected (ex 485 nm, em 535 nm) using the EnSpire (PerkinElmer). The amount of internalized probe was observed by comparing the fluorescence intensity associated with the cells before and after back-extraction.

### Detection of RBC adhesion to HUVECs

HUVECs were maintained in a 75 T flask with the EGM kit at 37 °C in a 95% O_2_ and 5% CO_2_ incubator. Endothelial cells (2 × 10^4^ cells) were seeded into 24 well plates for 3 days, and the medium was changed once the day after seeding. After 3 days, the endothelial cells were stained with calcein green for 20 min under dark conditions. After treatment of human RBCs (5 × 10^7^ cells/mL) with PS-NPs for 3 h, the RBCs were centrifuged and resuspended in EBM-2 to wash out PS-NPs. The resuspended RBCs were then added to calcein-loaded endothelial cells and incubated for 10 min. After incubation, the wells were washed with Hanks’ Balanced Salt Solution to remove unattached RBCs, and glycophorin A-PE was added for 10 min to label the RBCs. The extent of RBC adhesion to HUVECs was monitored using fluorescence microscopy (Eclipse Ti, Nikon). The fluorescence intensity of the adhered RBCs was analyzed using ImageJ software.

### Thrombin generation assay

After incubation with amine-modified polystyrene at 100 nm (PS-NP) for 3 h, RBCs (2.5 × 10^5^ cells) were incubated with 5 nM of factor Xa and 10 nM of factor Va in Tyrode buffer (134 mM of NaCl, 10 mM of HEPES, 5 mM of glucose, 2.9 mM of KCl, 1 mM of MgCl_2_, 12 mM of NaHCO_3_, 0.34 mM of Na_2_HPO_4_, 0.3% of BSA, and 2 mM of CaCl_2_ at pH 7.4) for 3 min at 37 °C. Thrombin formation was initiated by the addition of 2 μM of prothrombin. Three minutes after prothrombin supplementation, an aliquot of the suspension was transferred to a new tube containing stop buffer (50 mM of Tris–HCl, 120 mM of NaCl, and 2 mM of EDTA at pH 7.9). Thrombin activity was measured using the chromogenic substrate S2238. We determined the rate of thrombin formation based on the change in absorbance at 405 nm by referring to a calibration curve generated with purified human thrombin.

### Measurement with isolated rat RBCs

Adult male Sprague–Dawley rats (Harlan: Koatech, Pyeongtaek, Korea) were used in the animal experiments under the approval of the Institutional Animal Care and Use Committee at Hanyang University (IACUC 2020-0200A). Male Sprague–Dawley rats (250–400 g) were anesthetized by intraperitoneal (i.p.) administration of urethane (1.25 g/kg). Blood was withdrawn from the abdominal vein, and RBCs were isolated according to the same procedure for the isolation of human RBCs. Isolated rat RBCs were incubated with PS-NPs for 3 h, after which the extent of hemolysis, PS exposure, and thrombin generation were determined as described above.

### Rat in vivo thrombosis models

Thrombus formation was observed using venous thrombosis models in rats, as previously described [88]. Male Sprague–Dawley rats (250–400 g) were anesthetized with urethane (1.25 g/kg, i.p.). Three hours after the intravenous injection of PS-NPs suspended in saline, the abdomen was opened surgically to induce venous thrombosis. After a careful incision was made, the vena cava was exposed. Thromboplastin was infused for 1 min to induce clot formation. Thrombus formation was induced by tying the proximal and distal ends with cotton thread, and blood stagnation in the vena cava was maintained for 15 min. The ligated vein segment was excised, and the vein was carefully opened to separate the blood clots. The separated clot was carefully wiped off to remove excess blood, immediately weighed, and photographed.

### Statistical analysis

All experimental values were expressed as the mean and standard error (SE). Statistical significance between groups was determined using Student’s t-test and one-way analysis of variance followed by Dunnett’s post hoc test. SPSS version 24 was used for the data analysis. In all analyses*, p values* < 0.05 were considered statistically significant.

## Supplementary Information

**Additional file 1: Fig. S1.** PS exposure, MV formation and thrombin generation of various polystyrene microplastics and nanoplastics (PDF).

## Data Availability

The datasets used and/or analysed during the current study are available from the corresponding author on reasonable request.

## Abbreviations

EC: Endothelial cell
EDTA: Ethylenediaminetetraacetic acid
FITC: Fluorescein isothiocyanate
GSH: Glutathione
HUVEC: Human umbilical vein endothelial cell
MP: Microplastic
NP: Nanoplastic
MV: Microvesicle
PM: Particulate matter
PS: Phosphatidylserine
PS-NP: Polystyrene nanoplastic
RBC: Red blood cell
SE: Standard error
SEM: Scanning electron microscopy
TEM: Transmission electron microscopy
