# Supplementary material for: Amine-modified nanoplastics promote the procoagulant activation of isolated human red blood cells and thrombus formation in rats
Source: Part Fibre Toxicol. 2022 Sep 14;19:60. doi: 10.1186/s12989-022-00500-y (PMC9472436; doi:10.1186/s12989-022-00500-y)
Supplement: Supplementary file 1 — Additional file 1: Fig. S1. PS exposure, MV formation and thrombin generation of various polystyrene microplastics and nanoplastics (PDF). [file 12989_2022_500_MOESM1_ESM.pdf]

Additional file 1

Nanoplastics promote the procoagulant activation of isolated human red blood cells and thrombus formation in rats

Eun-Hye Kim[1], Sungbin Choi[1], Donghyun Kim[1], Han Jin Park[1], Yiying Bian[2], Sang Ho Choi[3], Han Young Chung[3,*] and Ok-Nam Bae[1,*]

[1]College of Pharmacy Institute of Pharmaceutical Science and Technology, Hanyang University, Ansan 15588, Republic of Korea

[2]School of Public Health, China Medical University, Shenyang, 110122, China

[3]National Research Laboratory of Molecular Microbiology and Toxicology, Department of Agricultural Biotechnology, and Center for Food and Bioconvergence, Seoul National University, Seoul 08826, Republic of Korea

Corresponding Authors:

Ok-Nam Bae, PhD

Email: onbae@hanyang.ac.kr; Tel: +82 31 400 5805

Han Young Chung, PhD

Email: robin302@snu.ac.kr; Tel: +82 2 880 4864

Total page : 2

Total number of tables : 0

Total number of figures : 1

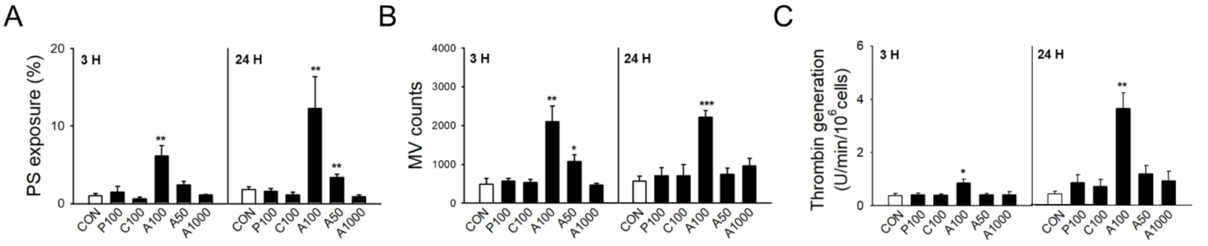

Figure. S1 PS exposure, MV formation and thrombin generation of various polystyrene microplastics and nanoplastics. We treated human RBCs with 100 µg/mL of amine-modified polystyrene 50 nm (A50), 100 nm (A100), and 1000 nm (A1000) in size; 100 nm plain polystyrene (P100); and 100 nm carboxyl-modified polystyrene (C100). The extent of externalization of phosphatidylserine (PS) (A), release of microvesicles (MVs) (B), and generation of thrombin (C) was analyzed at 3 h or 24 h after treatment of polystyrene particles to RBCs (n = 4 -6). Data are presented as the mean ± SE. * $p < 0.05$, ** $p < 0.01$, *** $p < 0.001$ vs. control (CON).
